# A systematic review and meta-analysis comparing combined intravenous and topical tranexamic acid with intravenous administration alone in THA

**DOI:** 10.1371/journal.pone.0186174

**Published:** 2017-10-10

**Authors:** Yangbai Sun, Chaoyin Jiang, Qingfeng Li

**Affiliations:** 1 Department of Plastic and Reconstructive Surgery, Shanghai Ninth People’s Hospital, Shanghai JiaoTong University School of Medicine, Shanghai, China; 2 Department of Orthopedic Surgery, Shanghai Sixth People’s Hospital, JiaoTong University, Shanghai, China; Cardiff University, UNITED KINGDOM

## Abstract

**Purpose:**

To compare the effectiveness and safety of combined intravenous and topical tranexamic acid with intravenous use alone in THA.

**Methods:**

The electronic databases MEDLINE, EMBASE, BIOSIS, Cochrane central, and further adapted for Google and Google Scholar internet, last updated on Dec 30, 2016, were searched. Evaluated outcomes included total blood loss, transfusion rate, maximum postoperative Hb drop, and incidence of thromboembolic complications. The standard mean difference (SMD) or the relative risk (RR) was calculated for continuous or dichotomous data respectively. The quality of the trial was assessed, and meta-analyses were performed with the Cochrane Collaboration’s RevMan 5.0 software.

**Results:**

Five RCTs with 457 patients were included. Combined TXA administration reduced blood loss (SMD, 1.39; 95%CI, 0.55 to 2.23; P<0.00001, I^2^ = 94%), hemoglobin decline (SMD, 0.84; 95%CI, 0.13 to 1.54; P = 0.01, I^2^ = 83%) and the need for transfusion (RR, 2.58; 95%CI, 1.59 to 4.18; P = 0.65, I^2^ = 0%) without increasing the rate of thromboembolic complications significantly (RR, 0.83; 95%CI, 0.27 to 2.54; P = 0.81, I^2^ = 0%).

**Conclusion:**

The present study has emphasized that combined TXA administration can effectively reduce blood loss, hemoglobin decline and the need for transfusion without increasing the rate of thromboembolic complications.

## Introduction

Total hip arthroplasty (THA) is one of the most common treatments in orthopedic surgery, associated with large amount of postoperative blood loss and significant rate of blood transfusion. It was estimated that the average blood loss during primary THA can be between 1000 and 2000 ml, and up to 37% of patients undergoing primary THA require blood transfusion for postoperative anemia[1]. Postoperative anemia may impede functional ability and increase the length of hospital stay and the costs. To reduce the need for blood transfusion, a variety of techniques have been used, including blood salvage, controlled hypotension, stimulation of erythropoiesis and the use of pharmacologic agents, such as Tranexamic acid[2].

Tranexamic acid (TXA), an antifibrinolytic drug which blocks the lysine blinding sites on plasminogen molecules and inhibits the formation of plasmin, has been widely used currently[3]. A large amount of evidences provided by prospective randomized controlled trials[4, 5] and meta-analysis studies[6, 7] have shown that TXA, applied either intravenously or locally, can reduce blood loss and subsequent transfusion without risking a high complication rate. Theoretically, a topical application of TXA may limit systemic absorption and decrease the risk of postoperative thromboembolic complications. It is proposed that topical application provides a maximum therapeutic concentration of TXA at the bleeding site, and induces partial microvascular hemostasis[8]. Another method which is the combined application of IV-TXA and topical TXA is deemed to be more effective than either regimen alone according to the above findings[9]. Recently, more and more researches have focused on the issue that whether combination application of intravenous and topical tranexamic acid has bigger value in THA when compared with only intravenous tranexamic acid[10–12].

Accordingly, we systematically reviewed the current evidences to investigate the effectiveness and safety associated with use of TXA by comparing combined intravenous and topical tranexamic acid with intravenous use alone. A Meta-analysis method was used to allow us to provide better evidence-based advice to researchers in this area.

## Materials and methods

This systematic review and meta-analysis was performed according to PRISMA Statement Criteria[13].

### Study selection and data extraction

The electronic databases MEDLINE, EMBASE, BIOSIS, Cochrane central, and further adapted for Google and Google Scholar internet, last updated on Dec 30, 2016, were searched. We reviewed the bibliographies of original trials, meta-analysis, and review articles identified for potential eligible articles. Moreover, gray literatures were also selected from the reference list of included studies. There were no restrictions as to the language and date. The search terms including ‘total hip arthroplasty’, ‘total hip replacement’, ‘THA’, ‘THR’, ‘tranexamic acid’ and ‘TXA’ were retrieved in the title, abstracts, and Medical Subject Headings. The search strategy was designed and refined, and two reviewers applied the search strategy to select references. In case of disagreement, it was discussed and consulted by a senior reviewer.

Two reviewers independently extracted all relevant data. Disagreement were resolved by discussion and, when necessary, with adjudication by a third reviewer. Effective data on the following study characteristics included: patient demographics, publication year, type of surgery, sample size, dose and administration of TXA, prophylactic antithrombotic therapy, surgical approach, blood transfusion trigger, total blood loss, Max Hb drop, transfusion rate, and incidence of thromboembolic complications. When necessary, details were sought from the authors of the primary studies.

### Inclusion and exclusion criteria

Inclusion criteria included (1) Randomized controlled trials (RCTs); (2) skeletally mature patients, aged 18 or older who received THA treatment regardless of the type or size of prosthesis implanted; (3) the intervention included intravenous and combined administration of TXA; (4) outcomes included total blood loss, transfusion rate, maximum postoperative Hb drop, and incidence of thromboembolic complications; (5) >10 patients in each group; (6) the primary outcome measures included transfusion rate, total blood loss, and maximum postoperative Hb drop; and the secondary outcome was the incidence of thromboembolic complications. Exclusion criteria included (1) patients with bleeding disorders or were on anticoagulant treatment; (2) animal models.

### Statistical analysis

Study data were pooled together and analyzed by the Cochrane Collaboration’s RevMan 5.0 software. For each study, relative risks (RR) and 95% confidence intervals (CIs) were calculated for dichotomous outcomes, and standard mean differences (SMD) and 95% confidence intervals (CIs) were calculated for continuous outcomes. A fixed-effect model with the inverse-variance test was used for continuous variables and the Mantel-Haenszel test for dichotomous variables when a lower heterogeneity was found; and a random-effect model was used when the heterogeneity was significant. The presence of statistical heterogeneity was assessed through Chi-square tests (with P < 0.05 representing heterogeneity) and I^2^ statistic (with I^2^ > 50% indicating high heterogeneity). Subgroup analysis was conducted to find the source if possible under significant heterogeneity. A sensitivity analysis was also performed by investigating the effect of each individual study on the pooled effect size.

### Assessment of methodological quality and publication bias

The methodological quality of these trials was evaluated in our meta-analysis. For RCTs, we assessed the risk of bias with the Cochrane Collaboration’s tool[14]. Assessments of five main fields included sequence generation, allocation concealment, blinding, incomplete outcome data and selective outcome reporting. The modified Jadad scale was applied to assess the quality of RCTs, and a score ≥ 4 was considered a high quality article[15].

Possible publication bias regarding to primary outcome transfusion rate was evaluated by the Begg’s rank correlation test and the Egger’s regression test. Moreover, funnel plot for blood transfusion rate was also generated to evaluate potential publication bias among studies. Both analyses are performed using STATA 10.0 software. All statistical tests were two-sided, and a P value <0.05 was considered statistically significant.

## Results

**Fig 1:**
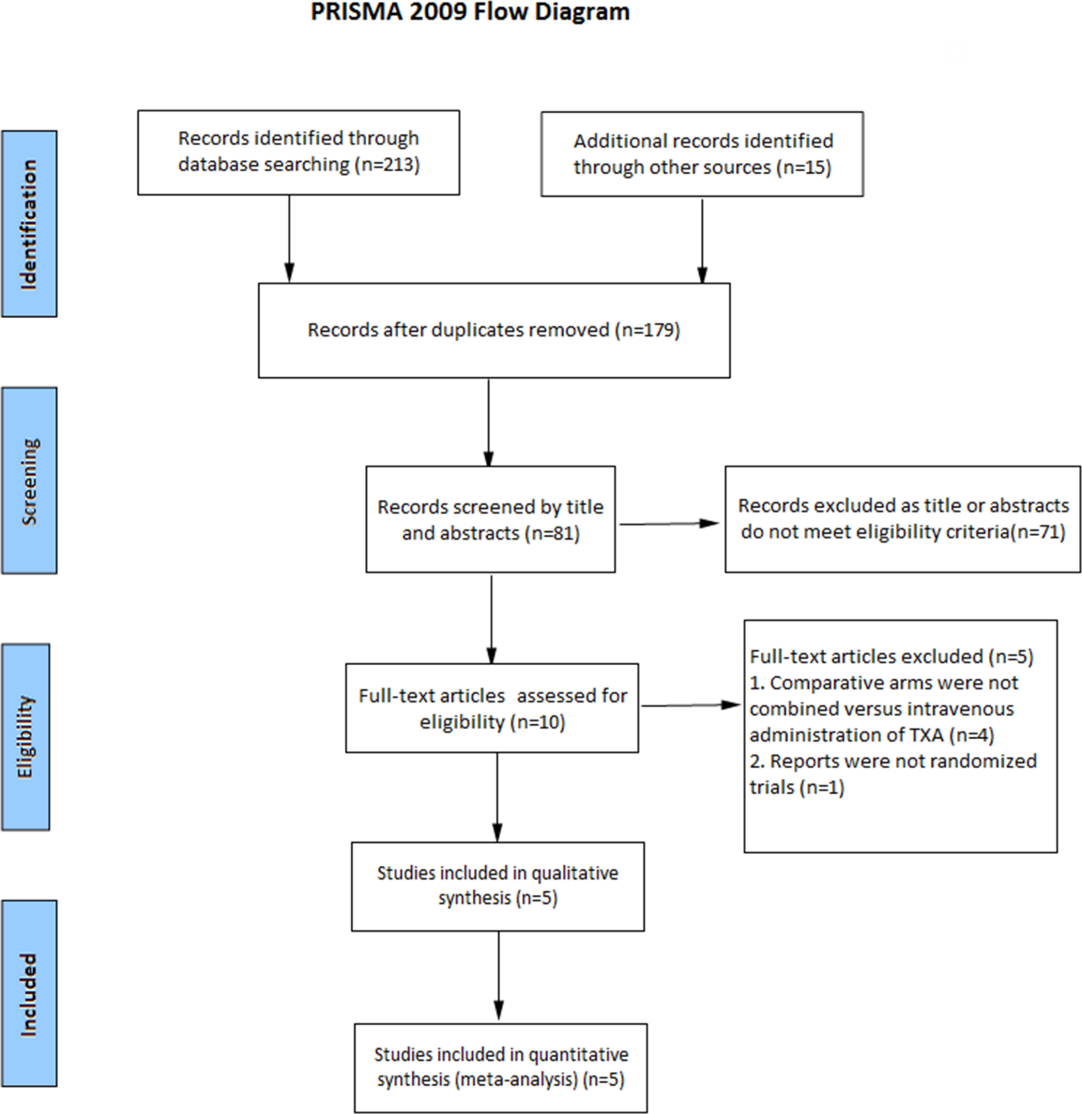
Flow chart.

A total of 228 potentially relevant articles were identified. After reference to titles, abstracts and even full texts, five RCTs eventually matched the inclusion and exclusion standard for analysis[10, 12, 16–18]. The search process was shown in Fig 1. Three studies were published in English[10, 12, 17], and two were published in Chinese[16, 18]. Table 1 showed the basal line of patients' characteristics. The sample size ranged from 65 to 140. Clinical outcomes of the included studies were documented in Table 2.

**Table 1:** Characteristics of included studies.

Studies	Surgery	TXA intervention	No.	Age	Sex ratio (M/F)	Prophylactic antithrombotic therapy	Surgical approach	Blood transfusion trigger	The modified Jadad scale
Xie 2015	Primary THA	I: 1.5g IV-TXA	70	59.5 ± 11.5	20/50	Enoxaparin	Posterolateral	Hb < 70g/L or Hb 70–100g/L with symptomatic anemia	6.5
		C: 1g IV-TXA+2g topical TXA	70	60.5 ± 11.0	22/28				
Wu 2016	Revision THA	I: 15mg/kg IV-TXA	42	59.5 ± 11.3	21/21	LMWH	Posterolateral	Hb < 80g/L or had symptoms of anemia	5
		C: 15mg/kg IV-TXA+3g topical TXA	42	60.1 ± 10.4	23/19				
Zeng 2016	Primary THA	I: 15mg/kg IV-TXA	50	54 ± 12.6	24/26	LMWH	Posterolateral	Hb < 70g/L or any anemia-related organ dysfunction	7
		C: 15mg/kg IV-TXA+1g topical TXA	50	53.6 ± 14.8	29/21				
Zhang 2015	Primary THA	I: 1g IV-TXA	34	63.4 ± 6.8	23/11	Enoxaparin	Anterolateral	Hb < 70g/L or Hb < 90g/L with anemia-related organ dysfunction	4
		C: 1g IV-TXA+100mg topical TXA	34	64.7 ± 8.1	24/10				
Sun 2016	Primary THA	I: 1g IV-TXA	34	66.2 ± 7.4	13/21	LMWH	Posterolateral	Hb < 70g/L	4
		C: 15mg/kg IV-TXA+1g topical TXA	31	64.8 ± 8.3	11/20				

I: Intravenous group; C: Combined group; LMWH: low-molecular-weight heparin.

**Table 2:** Outcomes of included studies.

Studies	TXA intervention	Blood loss (ml)	Max Hb drop	Transfusion rate	Incidence of thromboembolic complications
Xie 2015	IV-TXA	878.0±210	3.4±0.8	3(4.3%)	1
	Combined TXA	776.8±189.0	3.0±0.8	0(0%)	2
Wu 2016	IV-TXA	1794.6 ± 162.6	4.5±0.7	19(45.2%)	0
	Combined TXA	1326.9 ±143.2	3.7±0.6	9(21.4%)	1
Zeng 2016	IV-TXA	1002.6 ± 366.8	NR	8(16%)	2
	Combined TXA	835.5 ± 343.5	NR	1(2%)	2
Zhang 2015	IV-TXA	1019 ± 137	NR	9(26.5%)	0
	Combined TXA	787 ± 110	NR	5(14.7%)	0
Sun 2016	IV-TXA	1150 ± 310	NR	9(26.5%)	2
	Combined TXA	780 ± 270	NR	3(9.7%)	1

NR: Not report; I: Intravenous group; T: Topical group

Five studies examined were evaluated using the modified Jadad scale (Table 1). The modified Jadad score ranged from 4 to 7. Methodological quality of the included studies was detailed in Table 3. Two studies[10, 12] did not report the method of sequence generation, while three studies[12, 16, 18] did not report the blinding. Two studies[10, 12] used sealed envelopes for allocation concealment. All included studies reported outcomes of more than 95% of participants. Besides, all the results of selective outcome reporting were unclear.

**Table 3:** Assessments of risk of bias of the randomized controlled trials.

Studies	Sequence generation	Allocation concealment	Blinding	Incomplete outcome data	Selective outcome reporting
Xie 2015	Unclear	Opaque sealed envelopes	Yes	Yes	Unclear
Wu 2016	Unclear	Sealed envelopes	Unclear	Yes	Unclear
Zeng 2016	A computer-generated randomization list	Unclear	Yes	Yes	Unclear
Zhang 2015	A random number table	Unclear	Unclear	Yes	Unclear
Sun 2016	A random number table	Unclear	Unclear	Yes	Unclear

**Fig 2:**
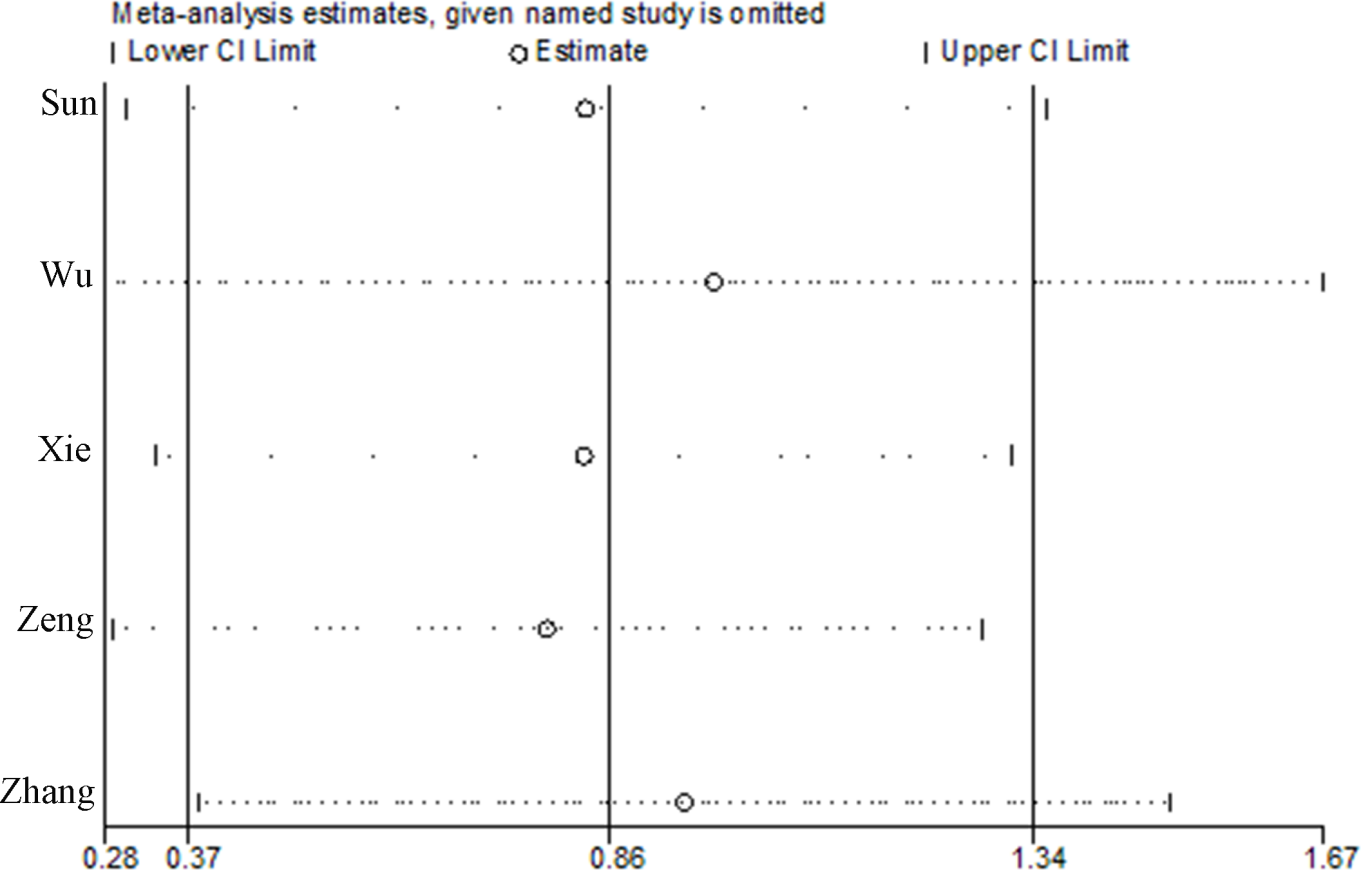
Sensitivity analysis of the meta-analysis.

**Fig 3:**
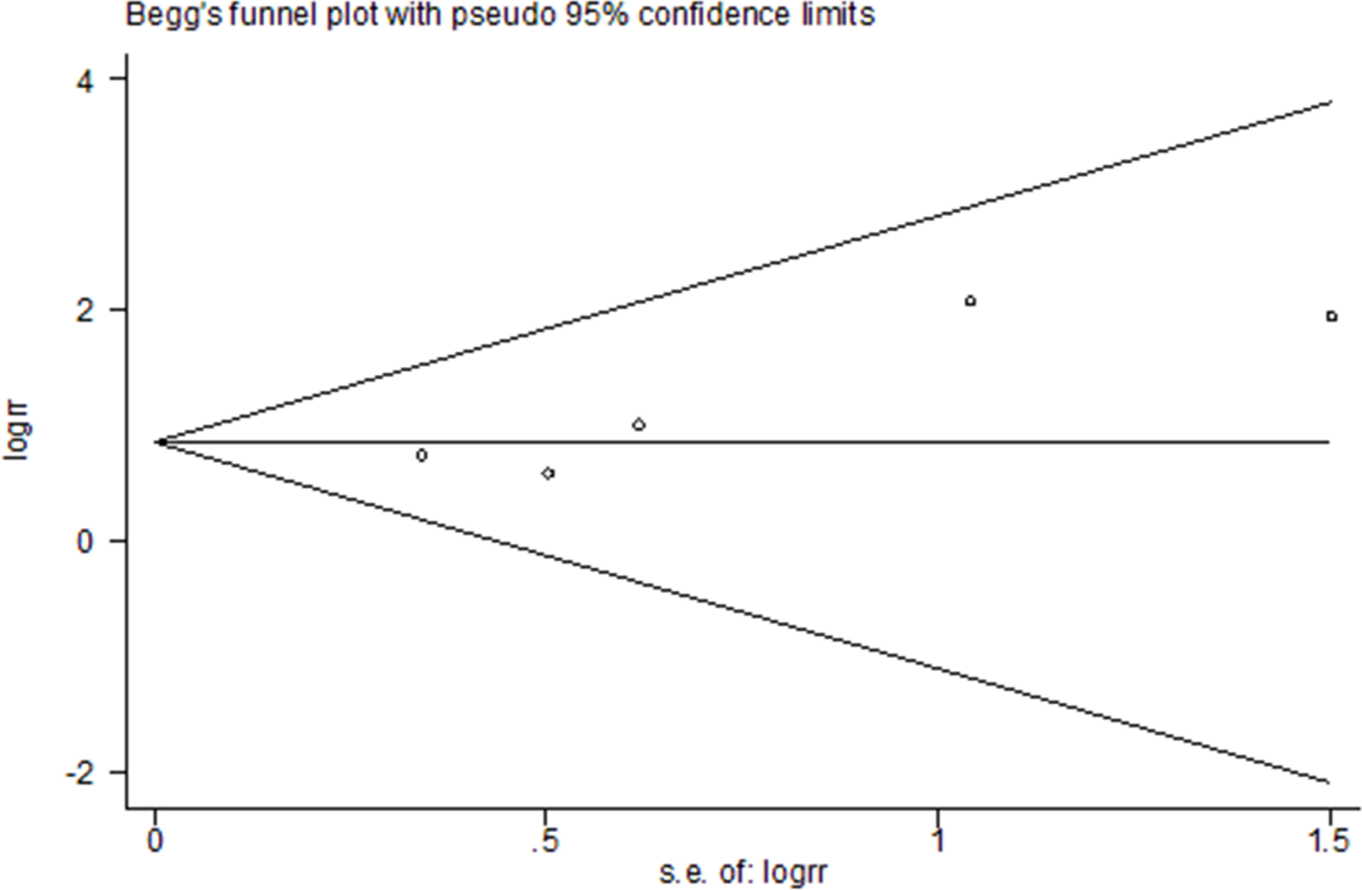
Publication bias analysis of the meta-analysis.

Sensitivity analyses were conducted by excluding each study one by one to explore whether one study markedly affected the results or highly contributed to the heterogeneity. It was indicated that the overall results were not substantially influenced by any single study and the data in this systematic review, and meta-analysis were in a relatively robust situation (Fig 2). There was little evidence of publication bias regarding to transfusion rate in relation to risk of intervention, as indicated by the Begg’s test (P = 0.221) and Egger’s test (P = 0.493). Fig 3 represented funnel plots examining for potential publication bias between studies.

**Fig 4:**
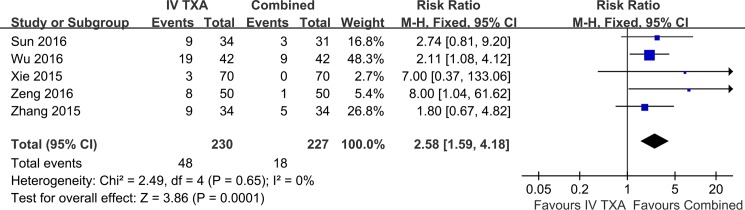
Meta-analysis of transfusion rate on IV-TXA versus combined TXA administration.

Five studies provided data on the effect of IV-TXA comparing combined TXA on blood transfusion in THA (Fig 4). The risk ratio for attaining blood transfusion was 2.58 (95%CI, 1.59 to 4.18) with applying the fixed-effects model. Results gave a pooled rate of 20.9% (48 of 230) in the IV-TXA group and of 7.9% (18 of 227) in the combined TXA group. IV-TXA administration led to a higher transfusion rate significantly. The studies consistently suggested benefit with little evidence of heterogeneity between findings (P = 0.65, I^2^ = 0%).

**Fig 5:**
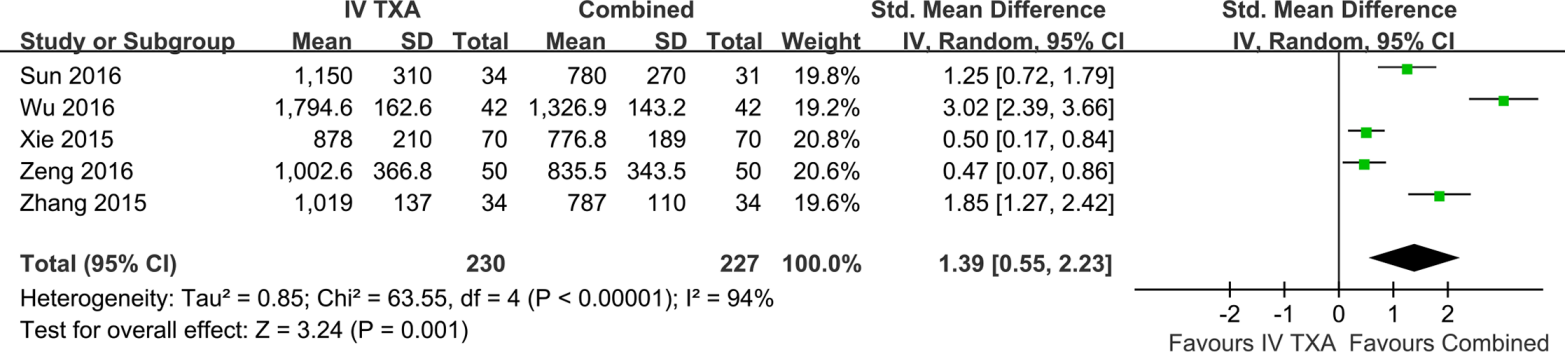
Meta-analysis of total blood loss on IV-TXA versus combined TXA administration.

Five studies reported on the amount of blood loss in THA (Fig 5). There was significant statistical heterogeneity between studies (P<0.00001, I^2^ = 94%) and random-effects was used. Combined TXA administration significantly reduced total blood loss in THA (SMD, 1.39; 95%CI, 0.55 to 2.23) compared with IV-TXA administration. Subgroup analysis was carried out for the amount of blood loss (Table 4). Perhaps the difference between primary and revision hip Arthroplasty was one of the reasons contributing to significant heterogeneity.

**Table 4:** Subgroup analysis relating total blood loss.

Group	No.	P _Heterogeneity_	I^2^	SMD	95%CI	P	P _Interaction_
Total	5	<0.00001	94%	1.39	0.55–2.23	0.001	
Surgery							
Primary THA	4	<0.0001	86%	0.98	0.39–1.58	0.001	<0.00001
Revision THA	1	-	-	3.02	2.39–3.66	<0.00001	
Surgical approach							
Posterolateral	4	<0.00001	95%	1.28	0.31–2.25	0.009	0.33
Anterolateral	1	-	-	1.85	1.27–2.42	<0.00001	
Antithrombotic therapy							
LMWH	3	<0.00001	96%	1.56	0.16–2.97	0.03	0.68
Enoxaparin	2	<0.0001	94%	1.15	-0.16–2.47	0.09	

**Fig 6:**
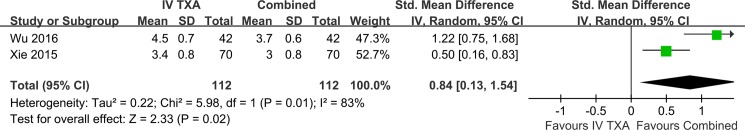
Meta-analysis of maximum postoperative Hb drop on IV-TXA versus combined TXA administration.

Two studies with 224 patients presented data on maximum postoperative Hb drop after surgery (Fig 6). There was significant statistical heterogeneity between studies (P = 0.01, I^2^ = 83%), and random-effects was used. Pooled results revealed higher maximum postoperative Hb drop in the IV-TXA group compared with combined group (SMD, 0.84; 95%CI, 0.13 to 1.54).

**Fig 7:**
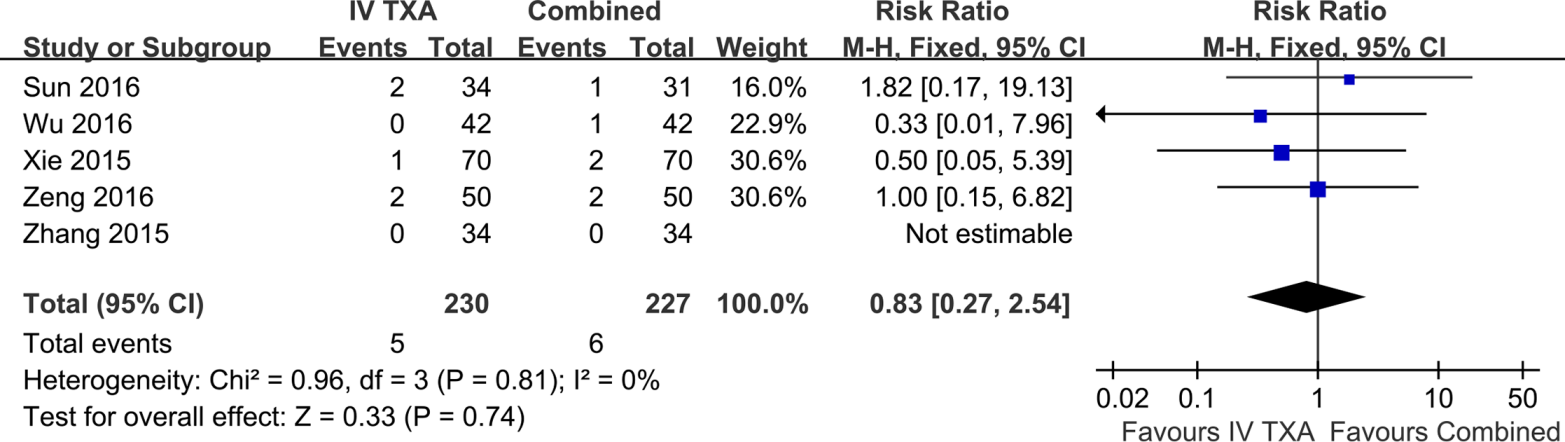
Meta-analysis of thromboembolic complications on IV-TXA versus combined TXA administration.

The data of thromboembolic complications were available in all five studies (Fig 7). It included intramuscular venous thrombosis, deep vein thrombosis and pulmonary embolism. There were 5 of 230 in the IV-TXA group and 6 of 227 in the combined TXA group. No significant difference was found between IV-TXA group and combined group (RR, 0.83; 95%CI, 0.27 to 2.54). There was no evidence of heterogeneity between studies (P = 0.81, I^2^ = 0%).

## Discussion

Five RCTs involving the application of TXA in THA fulfilled the inclusion criteria of this meta-analysis. Combined intravenous and topical TXA administration reduced blood loss, hemoglobin decline and the need for transfusion without increasing the rate of thromboembolic complications significantly.

Five RCTs met the inclusion criteria of meta-analysis and have all been published since 2015. The quality of each study was graded and scored from 4 to 7 according to the modified Jadad scoring system. Two studies[10, 12] did not report the method of sequence generation, while three studies[12, 16, 18] did not report the blinding. Two studies[10, 12] used sealed envelopes for allocation concealment. All five studies about selective outcome reporting were unclear. These issues may have resulted in low modified Jadad scores and information bias. Higher quality RCTs is needed for better assessing the effect of TXA administration methods. Pooled results showed significant heterogeneity when analyzing the amount of blood loss, so subgroup analysis was performed. The difference between primary and revision hip Arthroplasty was one of the reasons contributing to significant heterogeneity. It may also result from variations in methodology, different types of prosthesis, or different doses of TXA, etc. However, there were insufficient data to support these subgroup analyses. In addition, we performed a sensitivity analysis by investigating the effect of each individual study on the pooled effect size, and it was indicated by the results that no article substantially influenced the results and the current meta-analyses were comparatively reliable. Although our results showed that combined TXA group was effective in reducing blood loss, pooling data from a range of surgical operations resulted in substantial statistical heterogeneity and uncertain clinical interpretation. In short, due to apparent heterogeneity across studies and a paucity of included studies, the findings from our study should be dealt with some caution.

In THA treatment, it may cause significant perioperative bleeding because of the large exposed surface of cancellous bone and activation of local fibrinolysis. Patients, with elder age, were often affected by postoperative anemia and associated with increasing mortality and morbidity. TXA was introduced with the aim of reducing blood loss and transfusion requirements.

Intravenous administration of TXA in THA has been well established in the literature. Theoretically, intravenous TXA was hypothesized to be distributed in the whole blood cycle, thereby reducing its therapeutic concentration at the bleeding site, while topically applied TXA was predominantly distributed in the bleeding site and supplied a higher concentration[19]. It was considered to have little or no systemic absorption of TXA when applied topically, with the advantage of preventing the potential thromboembolic complications[20]. Chang reported a smaller decrease in hemoglobin (1.87 g/dL) and a lower transfusion rate (17% versus 35%) in the topical TXA group, and no increase in major complications was identified in patients managed with topical tranexamic acid[21]. Previous systematic reviews and meta-analyses have discussed the efficacy of topical and intravenous regimen of tranexamic acid in primary total hip arthroplasty[22–24]. The topical administration of TXA in THA carried similar hemostasis effects compared with intravenous use without an increased risk of thrombotic complications[22]. However, the optimal route for TXA application remains unknown. Recently, another mode of TXA administration which combined the use of intravenous and topical TXA has been introduced in THA. It was deemed to be more effective than either regimen alone. Nielsen et al[25] reported a persistent and sustained reduction in blood loss of 37% for combined IV and IA TXA compared with IV-TXA alone, at 24 hours postoperatively and on postoperative day 2. As related studies were too lacking, the effect of combined intravenous and topical TXA administration was unclear, when compared with intravenous or topical administration alone.

In 2016, two meta-analyses[26, 27] comparing combined use of intravenous and topical versus intravenous tranexamic acid in primary total joint arthroplasty was reported. However, both total knee arthroplasty and total hip arthroplasty were included in these studies. Of note, a feature specific to TKA was the common use of a tourniquet, which would help reducing local bleeding before TXA administration and is likely to impair TXA delivery to the surgical site intra-operatively[28]. This change may contribute to the increasing heterogeneity between two procedures. In our meta-analysis, this was the first time comparing IV-TXA with combined TXA administration in THA. We reviewed the clinical data currently available and tried to answer the question of the most effective regimen for TXA administration. In this study, the pooled rate of transfusion in the combined TXA group was 7.9% compared with 20.9% in the IV-TXA group, suggesting that combined TXA group significantly reduced the risk of transfusion. In addition, total blood loss and maximum Hb drop in the combined TXA group were significantly decreased when comparing with intravenous TXA studies. Therefore, combined TXA administration was clinically superior to IV-TXA in reducing blood loss, hemoglobin decline and the need for transfusion.

Zeng et al[17] reviewed previously reported results of IV and topical administration of TXA in THA. Most surgeons preferred a 10–30 mg/kg or 1–2g dose of TXA for IV use and 1 or 3 g for topical use. Ueno[29] found that a lower amount of TXA with IV administration can be used to achieve appropriate outcomes in THA than topical TXA administration. However, it was recommended for a single-TXA-use strategy (intravenous or topical), and the dose for combined administration was still unclear[17]. In our meta-analysis, all studies reported the dose for combined TXA administration. It was a 15mg/kg or 1g dose of TXA for IV use and a 0.1g-3g dose for topical use. Except Wu 2016 for revision THA, it was feasible and safety, with a total dose of TXA of approximately 3 g or less for combined administration. Rajesparan et al[30] reported a negative correlation between the dose per kg of TXA and total blood loss in women. However, the number of patients in the study was limited. The maximum dose of TXA was approximately 3 g for primary THA in some literatures[28].

In our meta-analysis, IV-TXA was administered 5–15 minutes before skin incision, and topical TXA was applied at the acetabulum and the femoral canal after acetabulum and femur preparation and the joint space after fascial closure. When combined TXA was administrated, the duration of fibrinolysis activation would be longer than intravenous use alone. In terms of equal total dose, it would maintain a high plasma concentration with combined TXA administration than the single intravenous use[10], which might be the reason that we found less blood loss in combined group.

The potential thromboembolic complications should also be concerned. So far, TXA has been applied in THA for more than ten years; a larger amount of clinical evidence had confirmed the safety, without increasing the incidence of both DVT and PE[31]. A retrospective study by Poeran et al[32] included more than 870, 000 patients receiving THA or TKA during a 6-year period, the results found no significant difference on postoperative DVT between TXA and control group (0.4 Vs 0.5%). In our meta-analysis, four studies except Zhang 2015 have reported that Doppler ultrasound was used to detect thromboembolic complications. All the studies used low molecular weight heparin (LMWH) or Enoxaparin as DVT prophylaxis. Total, five cases of thromboembolic complications (2.2%) was found in IV-TXA group and six cases (2.6%) were in the combined TXA group, which did not reach a significant statistical difference. It was indicated that there was no superiority in the rate of thromboembolic events in combined TXA group compared with intravenous TXA group in this study. Nonetheless, uncertainty still remains as the difference of intramuscular venous thrombosis, and DVT and PE screening and more evidence were required to assess the safety of TXA[8].

There are limitations to the current analysis. Firstly, only five reports were included, and the sample size of each trial was small, which would influence the results. Secondly, most of the available data would support the safety in healthy patients for TXA use rather than those with higher risks. It was underpowered, making it difficult to draw reliable conclusions regarding the use of TXA in high-risk patients[2]. Finally, the optimal dose of TXA administration was still unclear. The decision was sometimes based on the surgeon’s preference or the experience from previous literatures. Additional large studies were required to further refine the right dose of TXA administration.

## Conclusion

The present study has emphasized that combined TXA administration can effectively reduce blood loss, hemoglobin decline and the need for transfusion without increasing the rate of thromboembolic complications when compared with IV-TXA use alone. It is the initial systematic review that evaluates the safety and efficiency of combined TXA administration versus intravenous use alone following THA. High-quality RCTs and well-designed trails are still needed to detect the therapeutic dose or other adverse effects in the future.

## Supporting information

S1 File PRISMA checklist. (PDF)

S2 File The search strategy. (DOC)
