# Supplementary material for: A systematic review and meta-analysis comparing combined intravenous and topical tranexamic acid with intravenous administration alone in THA
Source: PLoS One. 2017 Oct 10;12(10):e0186174. doi: 10.1371/journal.pone.0186174 (PMC5634626; doi:10.1371/journal.pone.0186174)
Supplement: S2 File — (DOC) [file pone.0186174.s002.doc]

The search string of our research

PubMed database

#1 Arthroplasty, Replacement, Hip [Mesh]

#2 Total Hip Arthroplasty [All Fields]

#3 Total Hip Replacement [All Fields]

#4 THA [All Fields]

#5 THR [All Fields]

#6 #1 OR #2 OR #3 OR #4 OR #5

#7 Tranexamic Acid [Mesh]

#8 Tranexamic Acid [All Fields]

#9 TXA [All Fields]

#10 #7 OR #8 OR #9

#11 #6 AND #10

EMABSE, BIOSIS, Cochrane central and Google Scholar internet

#1 Total Hip Arthroplasty

#2 Total Hip Replacement

#3 THA

#4 THR

#5 #1 OR #2 OR #3 OR #4

#6 Tranexamic Acid

#7 TXA

#8 #6 OR #7

#9 #5 AND #8
